# 5-Hydroxymethylcytosine Is Not Present in Appreciable Quantities in *Arabidopsis* DNA

**DOI:** 10.1534/g3.114.014670

**Published:** 2014-11-06

**Authors:** Robert M. Erdmann, Amanda L. Souza, Clary B. Clish, Mary Gehring

**Affiliations:** * Whitehead Institute for Biomedical Research, Cambridge, Massachusetts 02142; † Department of Biology, Massachusetts Institute of Technology, Cambridge, Massachusetts 02139; ‡ Broad Institute, Cambridge, Massachusetts 02142

**Keywords:** DNA demethylation, *Arabidopsis*, 5-hydroxymethylcytosine, 5-methylcytosine, epigenetics

## Abstract

5-Hydroxymethylcytosine (5-hmC) is an intermediate in active demethylation in metazoans, as well as a potentially stable epigenetic mark. Previous reports investigating 5-hydroxymethylcytosine in plants have reached conflicting conclusions. We systematically investigated whether 5-hmC is present in plant DNA using a range of methods. Using the model organism *Arabidopsis thaliana*, in addition to other plant species, we assayed the amount or distribution of 5-hydroxymethylcytosine by thin-layer chromatography, immunoprecipitation-chip, ELISA, enzymatic radiolabeling, and mass spectrometry. The failure to observe 5-hydroxymethylcytosine by thin-layer chromatography established an upper bound for the possible fraction of the nucleotide in plant DNA. Antibody-based methods suggested that there were low levels of 5-hmC in plant DNA, but these experiments were potentially confounded by cross-reactivity with the abundant base 5-methylcytosine. Enzymatic radiolabeling and mass spectrometry, the most sensitive methods for detection that we used, failed to detect 5-hydroxymethylcytosine in *A. thaliana* genomic DNA isolated from a number of different tissue types and genetic backgrounds. Taken together, our results led us to conclude that 5-hmC is not present in biologically relevant quantities within plant genomic DNA.

Plants have robust, well-characterized systems for the deposition, maintenance, and removal of DNA methylation (Law and Jacobsen 2010). The presence of 5-methylcytosine (5-mC) is correlated with reduced transcription when present in promoters or transposable elements (Mette *et al.* 2000; Kato *et al.* 2003). In plants, 5-mC is found in all sequence contexts and ranges from 5% to 30% of cytosines depending on the species (Wagner and Capesius 1981; Leutwiler *et al.* 1984). Removal of 5-methylcytosine plays key roles in plant reproductive development and in counteracting excessive DNA methylation (Zhu 2009; Gehring *et al.* 2009b). *Arabidopsis thaliana* has four 5-methylcytosine DNA glycosylase enzymes involved in the removal of DNA methylation by base excision repair: ROS1, DML2, DML3, and DME (Zhu 2009). DME is required for DNA demethylation in one of the female gametes before fertilization, a process that is essential for normal seed development (Gehring *et al.* 2006), and the other 5-methylcytosine DNA glycosylases prevent methylation from accumulating near genes during vegetative development (Penterman *et al.* 2007; Lister *et al.* 2008). DME and ROS1 excise 5-mC and T from T:G mismatches *in vitro* (Gehring *et al.* 2006; Morales-Ruiz *et al.* 2006) and have very recently been shown to also excise 5-hmC, although less efficiently than 5-mC (Jang *et al.* 2014).

5-hydroxymethylcytosine (5-hmC) was first reported in bacteriophage DNA in 1952 (Wyatt and Cohen 1952) and was observed in animal brain and liver genomic DNA in 1972 (Penn *et al.* 1972). Its presence in mammalian genomes was confirmed by a pair of research groups in 2009 (Kriaucionis and Heintz 2009; Tahiliani *et al.* 2009; Branco *et al.* 2012; Kohli and Zhang 2013). There is no homolog to the plant 5-methylcytosine DNA glycosylase enzymes in metazoans, yet loss of DNA methylation occurs at critical stages of animal development. Thus, it was hypothesized that 5-hmC might be involved in the masking and/or the removal of 5-mC from the DNA of animals. The Ten Eleven Translocation (TET) family of enzymes is responsible for the oxidation of 5-mC into 5-hmC and successive oxidation to 5-formylcytosine and 5-carboxylcytosine (Tahiliani *et al.* 2009; Ito *et al.* 2011). Embryonic stem cells lacking the three TET enzymes are compromised in their ability to differentiate and exhibit promoter hypermethylation (Dawlaty *et al.* 2014). Increases in 5-hydroxymethylation are found in the same early embryonic mouse tissues where 5-methylcytosine levels are known to decline during developmental epigenetic reprogramming (Iqbal *et al.* 2011). 5-hmC has also been implicated as a unique epigenetic mark aside from its role as a demethylation intermediate. In mouse nervous tissues, 5-hmC is enriched in actively transcribed genes and is bound by MeCP2 (METHYL-CPG-BINDING PROTEIN 2), a protein that is associated with transcriptional repression when bound to 5-mC (Mellén *et al.* 2012).

One of the most widely used methods for the analysis of genome-wide 5-mC distribution, bisulfite sequencing, relies on the differential reactivity of sodium bisulfite toward cytosine and 5-mC. However, 5-hmC and 5-mC are indistinguishable by bisulfite sequencing (Nestor *et al.* 2010; Huang *et al.* 2010; Jin *et al.* 2010). Therefore, 5-mC identified through this method actually represents some mixture of 5-mC and 5-hmC in genomes that contain both bases in appreciable quantities. Other recently developed methods offer the ability to distinguish between 5-hmC and 5-mC while sequencing. Oxidative bisulfite sequencing consists of the selective oxidization of 5-hmC to 5-formylcytosine prior to bisulfite conversion (Booth *et al.* 2012). Tet-assisted bisulfite sequencing protects 5-hmC residues through glycosylation prior to Tet-mediated conversion of 5-mC to 5-carboxylcytosine and subsequent bisulfite treatment (Yu *et al.* 2012). However, bisulfite sequencing is widely used in plant epigenetics research, and thus it is imperative to ensure that the methylation patterns reported are fully 5-mC in origin. Although presence of 5-hmC in plants is not necessary to explain demethylation dynamics in the genome, it could be part of a complementary demethylation pathway or act as an independent epigenetic mark. However, there are no known homologs of TET enzymes within *Arabidopsis* or other plants (Iyer *et al.* 2009), meaning if that 5-hmC was present in the genome, then the mechanism of its production would be uncharacterized.

5-hmC was reported in barley aleurone tissue using two-dimensional paper chromatography in 1977 (Taiz and Starks 1977). More recent studies investigating the presence or absence of 5-hmC in plants have reached conflicting conclusions. Some have argued that it is present in measurable quantities (Yao *et al.* 2012), or that related further oxidized derivatives are present (Tang *et al.* 2014). Others have argued that there is no evidence for its presence (Jang *et al.* 2014), or that it is present in trace quantities that are unlikely to be biologically relevant (Liu *et al.* 2013). Here, we characterize the level of 5-hmC primarily in the genome of *Arabidopsis thaliana* using a wide range of experimental methods. We conclude that 5-hmC is not present in *Arabidopsis* DNA above trace levels. We also argue that some methods for 5-hmC detection may not be ideal for use in situations when 5-hmC is not present in high concentrations.

## Materials and Methods

### Plant material and DNA isolation

All plants were grown in 16 hr of light per day in a growth room maintained at approximately 21**°**. DNA was isolated using either the DNeasy Plant Mini or Maxi kit (Qiagen).

### Synthetic DNA controls

Control DNA containing 5-hmC, mC, or C was amplified by PCR using a dNTP mixture containing dATP, dTTP, dGTP, and dCTP (Invitrogen), dm5CTP (New England BioLabs), or dhmCTP (Bioline). Control DNA for ELISAs, TLC, mass spectrometry, and β-glucosyltransferase labeling was PCR amplified from *Saccharomyces cerevisiae* genomic DNA using oligos to *FLRI* (FLR1-F: 5′-TTTGTGGGTTCTCAGGATCAC; FLR1-R: 5′-TCCTCTACCGATTCAGACTCTAG), an 1164-bp region with 41% GC content, and *HOPI* (HOP1-F: 5′-GTCACGGGCATTTGTAGTTGCG; HOP1-R: 5′-TTAACACGGTGGCTGCTTTGGG), a 1074-bp region with 39% GC content. PCR products were run on an agarose gel and isolated using a QIAquick Gel Extraction kit (Qiagen). DNA controls for slot blot experiments were amplified from a 552-bp region of *A. lyrata* DNA containing 21 cytosines.

### Thin-layer chromatography

1 μg of Col-0 10-d-old shoot DNA or synthetic control DNA was incubated with 50 units of *Taq*^α^I and 100 μg of RNase A at 60**°** for 1 hr; 2.5 units of shrimp alkaline phosphatase were added and samples were incubated at 37**°** for 30 min. DNA fragments were isolated using a QIAquick Nucleotide Removal kit (Qiagen). End labeling of DNA fragments was performed with T4 polynucleotide kinase and 4 μCi γ^32^P ATP. DNA fragments were separated from unincorporated nucleotides using Illustra MicroSpin G-25 columns (GE Healthcare), ethanol precipitated, and then treated with 100 milliunits of snake venom phosphodiesterase I (SVPD) (Worthington) and 50 units of Benzonase (Novagen) for 2 hr at 37**°** before heat inactivation at 65**°** for 20 min. Polyethyleneimine-cellulose precoated glass plates (EMD Millipore) were spotted with 2.4 μL (100 ng) of the digested DNA. Plates were placed in glass TLC chambers with a solution of 62.5% isobutyric acid, 33.75% deionized water, and 3.75% ammonium hydroxide for the first dimension. After 20 hr, the plates were removed and dried. The plates were rotated 90 degrees counterclockwise and placed in a solution of 70% isopropanol, 15% hydrochloric acid (37%), and 15% deionized water. The plates were removed and dried after 20 hr and then exposed to storage phosphor screens, which were imaged using a Typhoon Phosphorimager (Amersham).

### Slot blots

Genomic DNA was isolated from *A. thaliana* (Col-0) and *A. lyrata* rosette leaves and *C. rubella* flowers and then RNase-treated. DNA was sheared to between 200 and 2000 bp using a bioruptor sonicator (Diagenode); 400 ng of plant genomic DNA and 20 ng of a control 552 bp DNA containing approximately 10 pmol of C, 5-hmC, or 5-mC was heated at 100**°** in 0.34 M NaOH/8.5 mM EDTA for 10 min, chilled on ice for 10 min, blotted onto a positively charged nylon membrane in duplicate using a slot blotter (Schleicher and Schuell), and cross-linked with UV. The membrane was cut, blocked in blocking solution (10% milk, 1% BSA, 1× PBS, 0.1% Tween-20) at 4**°** overnight, and then incubated with a 1:500 dilution of a rat monoclonal 5-hmC antibody (MAb-633HMC-100; Diagenode) for 1 hr at 4**°** or a 1:500 dilution of a mouse monoclonal 5-mC antibody 33D3 (Diagenode). The blots were washed for 10 min in 1× PBS/0.1% Tween-20 four times, incubated with a 1:50,000 dilution of goat α-rat HRP (horseradish peroxidase) (for α-5-hmC) or a 1:2000 dilution of sheep α-mouse HRP (for α-5-mC) in blocking solution for 1 hr at room temperature, and then washed as before. The membranes were incubated with SuperSignal West Dura extended duration substrate (ThermoScientific) for 5 min as directed and then exposed to film for 15 min (α-5-hmC) or 25 min (α-5-mC).

### IP-chip

Genomic DNA was isolated from Col-0 flower buds and open flowers and RNase-treated. 8.5 μg of DNA was sheared to between 200 and 1200 bp using a bioruptor (Diagenode); 4 μg (40 μL) of sheared DNA was added to each of two tubes. DNA was denatured by heating at 100**°** for 10 min and then quickly chilled in an ice-water bath for another 10 min. DNA for 5-hmC IP was diluted in 452 μL cold 1× meDIP buffer (10 mM sodium phosphate pH 7, 140 mM NaCl, 0.05% Triton X-100) (Weber *et al.* 2005) and 8 μL (10 μg) of α-5-hmC rat monoclonal antibody was added (MAb-633HMC; Diagenode). DNA for 5-mC IP was diluted in 440 ml cold 1× meDIP buffer and 20 μl (10 μg) of α-5-mC mouse monoclonal antibody 33D3 (Eurogentec) added. Tubes were rotated at 4**°** for 2 hr; 40 μL of Protein G Dynabeads or 40 μL of sheep α-mouse IgG Dynabeads (Invitrogen) were washed, resuspended in 1× meDIP buffer, and added to the 5-hmC and 5-mC IP, respectively, and tubes were rotated for 2.5 hr. Beads were then washed 3× for 10 min with 700 μL 1× meDIP buffer at room temperature, suspended in ProK digestion buffer (50 mM Tris pH 8, 10 mM EDTA, 0.5% SDS), and digested with 70 units of Proteinase K for 1 hr in a 55**°** water bath. DNA was extracted with phenol/chloroform/isoamyl alcohol and chloroform, ethanol-precipitated, and resuspended in water. Immunoprecipitated and input (sheared genomic DNA prior to IP) DNA was amplified using a whole genome amplification kit (WGA2) as described in the protocol (Sigma), except that 100 ng gDNA was used for input starting material. Amplified input and immunoprecipitated DNA was labeled with Cy3 and Cy5, respectively, as described (Deal and Henikoff 2010). A single replicate was performed. Labeled IP and input DNAs were cohybridized as described (Deal and Henikoff 2010) to a RocheNimblegen *Arabidopsis* high-density tiling array (Bernatavichute *et al.* 2008) at the Fred Hutchinson Cancer Research Center Genomics Shared Resource Laboratory.

### IP-qPCR

Immunoprecipitation (IP) was performed as described for IP-chip, except that in the first experiment 1.6 μg of DNA was immunoprecipitated with 4 μg of α-5-hmC rat monoclonal antibody (MAb-633HMC, Diagenode) or 4 μg mouse monoclonal α-5-mC antibody 33D3 (Diagenode). In the second experiment, 4 μg of DNA was immunoprecipitated with 10 μg of α-5-hmC rat monoclonal antibody (MAb-633HMC; Diagenode), 10 μg α-5-hmC rabbit polyclonal antibody (Active Motif), or 10 μg mouse monoclonal 5-mC antibody 33D3 (Diagenode). Regions of interest were amplified using Fast Sybr-Green mix (Applied Biosystems) according to manufacturers’ instructions. All reactions were performed using a StepOne Plus Real-Time PCR system (Applied Biosystems). Regions corresponding to AT1G15190 (MG519, 5′-AGATTCGACGGCACTGGCAAAG and MG520, 5′-AAAGGCGAAGTAAGGCTCTGGTG), AT1G57820 (MG521, 5′-TGCCAGATTGCTCACTCGAACAC and MG522, 5′-AGAAGCACACGGCGATCACTTG), AT1G22500 (MG523, 5′-ATTGATGCCTGGCTCCGTTCTC and MG524, 5′-ACCCGGTACAGGAACGAGATTG), and AT1G13410 (MG525, 5′-AGGTGGACATTGGCGAAGTTGC and MG526, 5′-AGCCGGGTTTCTTGGTTCAAGC) were amplified.

### ELISAs

ELISAs (enzyme-linked immunosorbent assays) were performed on 50–100 ng of genomic DNA using the MethylFlash Hydroxymethylated DNA Quantification Kit – Fluorometric (Epigentek) as directed. The plate was incubated for 2 min in the dark following the addition of fluoro-development solution before measurement in a TECAN SAFIRE II fluorescence microplate reader.

### β-glucosyltransferase radiolabeling

The method of Szwagierczak *et al.* (2010) was utilized with minor modifications; 500 ng of genomic DNA were used per glucosylation reaction. To improve detection for low concentration ranges, reactions contained 4 nM “cold” UDP-glucose (Sigma Aldrich), increasing the ratio of UDP-[6-^3^H]-glucose (Perkin-Elmer) to “cold” UDP-glucose. Glucosylation reaction incubation time was increased to 30 min. Radioactivity was measured using a Tri-Carb 3110TR Liquid Scintillation Analyzer (Perkin-Elmer), with 15-min measurements per sample.

### Mass spectrometry

To digest genomic DNA to single nucleosides, 1 μg was incubated with 10 units of Benzonase, 15 milliunits of SVPD, and 4 units of calf intestinal alkaline phosphatase (New England Biolabs) in 20 mM MgCl_2_ and 20 mM Tris solution at pH 8 for 12 hr at 37**°**. Following digestion, the solution was applied to Amicon Ultra 3000 molecular weight cut-off filters (Millipore) and spun at 21,000 RCFs for 30 min. The resulting solution was diluted 10-fold in a solution of 75% acetonitrile and 25% methanol. Liquid chromatography tandem mass spectrometry data were acquired using an 1100 Series pump (Agilent) and an HTS PAL autosampler (Leap Technologies) interfaced with a 4000 QTRAP triple quadrupole mass spectrometer (AB SCIEX). Nucleoside extracts (10 µL) were injected onto a 150- × 2.1-mm, 3-µm particle size Atlantis HILIC column (Waters). The column was eluted isocratically at a flow rate of 250 µL/min with 5% mobile phase A (10 mM ammonium formate and 0.1% formic acid in water) for 1 min, followed by a linear gradient to 40% mobile phase B (acetonitrile with 0.1% formic acid) over 10 min. Multiple reaction monitoring was used to measure nucleosides using the following transitions: 2′-deoxycytidine — 228.1 → 112.1 amu; 5-methyl-2′-deoxycytidine — 242.2 → 126.1 amu; 5-hydroxymethyl-2′-deoxycytidine — 258.2 → 142.1 amu. The ion spray voltage was 4.5 kV and the source temperature was 450°. MultiQuant 2.1 software (AB SCIEX) was used for peak integration and the data were manually reviewed for quality of integration and compared against a known standard to confirm identities.

## Results

### 5-hmC is not detected in *Arabidopsis* by TLC

**Figure 1:**
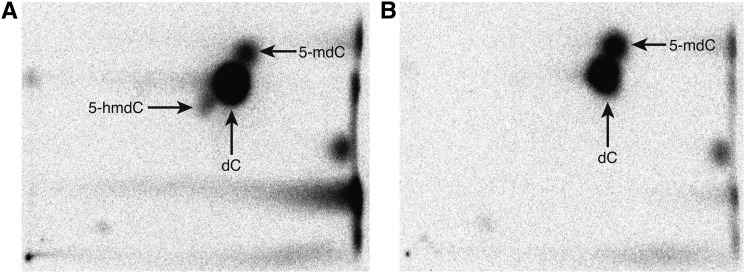
5-hmdC is not detected in *Arabidopsis* genomic DNA by TLC. (A) Control plate spotted with 100 ng mixture of 95% dC, 4% 5-mdC, and 1% 5-hmdC synthetic DNA. (B) Plate spotted with 100 ng of Col-0 10-d-old shoot DNA.

We performed two-dimensional thin-layer chromatography (TLC) to separate different forms of cytidine within DNA. Control plates spotted with a 100-ng mixture of 95% dC, 4% 5-mdC, and 1% 5-hmdC–containing synthetic DNA exhibited distinct spots for each of the three forms of cytidine (Figure 1A). TLC separation of 100 ng of digested *A. thaliana* genomic DNA from Col-0 shoots yielded deoxycytidine and 5-mdC spots, but no 5-hmdC spot was discernible (Figure 1B), consistent with results reported previously (Jang *et al.* 2014). We performed a dilution series of plates spotted with DNA mixtures containing 5-hmdC in percentages ranging from 5% to 0.01% of cytidines to determine the sensitivity of our TLC protocol. The threshold of detection was between 0.5% and 0.1%, at which point the 5-hmdC spot was no longer visually distinct from the outer area of the dC spot (Supporting Information, Figure S1). Thus, if 5-hydroxymethylcytidine is present in *Arabidopsis* DNA, it is less than 0.5% of all cytidines. This is below the level of 5-hmdC observed in mammalian cortex tissues (∼1.25%), but some tissue types, such as embryonic stem cells, have 5-hmdC levels (0.15%–0.3%) near our detection threshold (Szwagierczak *et al.* 2010; Le *et al.* 2011).

### Antibody-based methods yield positive results for 5-hmC

**Figure 2:**
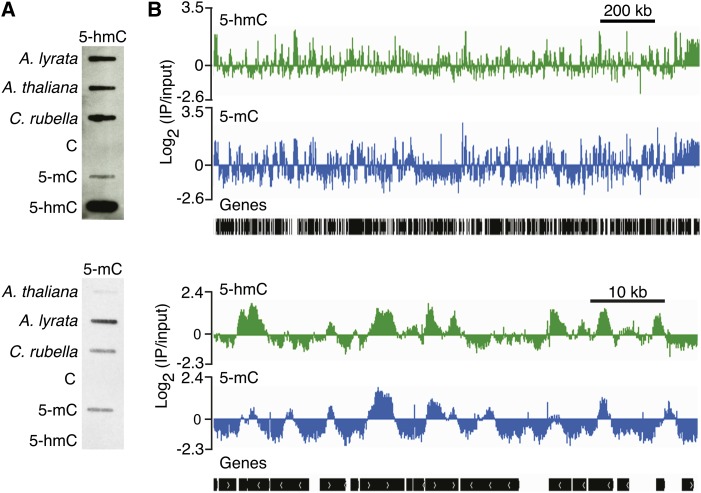
A 5-hmC antibody recognizes plant DNA. (A) Slot blot of 400 ng of genomic DNA from the indicated species and 20 ng of synthetic DNA containing 10 pmol of C, 5-mC, or 5-hmC as controls probed with a 1:500 dilution of a rat monoclonal 5-hmC antibody (top) or a 1:500 dilution of a mouse monoclonal 5-mC antibody (bottom). (B) IP-chip of 5-hmC (green track) and 5-mC (blue track) from *A. thaliana* genomic DNA. A 2-Mb representative region on chromosome 1 from 7000000 to 9000000 (top) and a 80-kb region from 5222870 to 5294010 (bottom) is shown.

DNA bases that occur at less than 0.5% of cytosines might still have functional significance. Thus, we performed a series of additional assays to determine if 5-hmC was present at low levels in plant DNA despite the lack of signal from TLC. Slot blots of genomic DNA from three closely related plant species, *A. thaliana*, *A. lyrata*, and *C. rubella*, probed with a mouse monoclonal 5-hmC antibody consistently yielded a robust signal (Figure 2A). The 5-hmC antibody also reacted against control DNA that contained 5-mC, although the signal was comparatively much weaker than for 5-hmC controls (Figure 2A).

To assess the global distribution of 5-hmC in comparison with 5-mC, we performed immunoprecipitation (IP) simultaneously from *A. thaliana* flower bud DNA with 5-hmC and 5-mC antibodies (Figure 2B). Immunoprecipitated DNA was amplified and hybridized to a high-density *Arabidopsis* tiling microarray. 5-hmC arises in DNA from oxidation of 5-mC, and thus 5-hmC might be expected to occur in regions with 5-mC. The 5-hmC and 5-mC profiles were correlated (Pearson’s *R* = 0.75) (Figure S2A). The majority of probes that were high in 5-hmC (log_2_ 5-hmC probe value ≥1) were also enriched for 5-mC (Figure S2B). However, we also observed regions with high 5-hmC values that were absent from 5-mC profiles, which could indicate that 5-mC can be fully and stably converted to 5-hmC in DNA (Figure 2B). 5-hmC levels for probes where log_2_ 5-mC values were ≥1 showed a greater degree of variability (Figure S2C). We performed independent IP experiments with two different 5-hmC antibodies as well as a 5-mC antibody to validate the whole genome profiling results by quantitative PCR (qPCR). The qPCR results generally agreed with the whole genome profiling; regions with high 5-mC and low 5-hmC were enriched in 5-mC IP, but not 5-hmC IP, and regions with high 5-hmC and low 5-mC were enriched in 5-hmC IP compared with 5-mC IP (Figure S2D).

**Figure 3:**
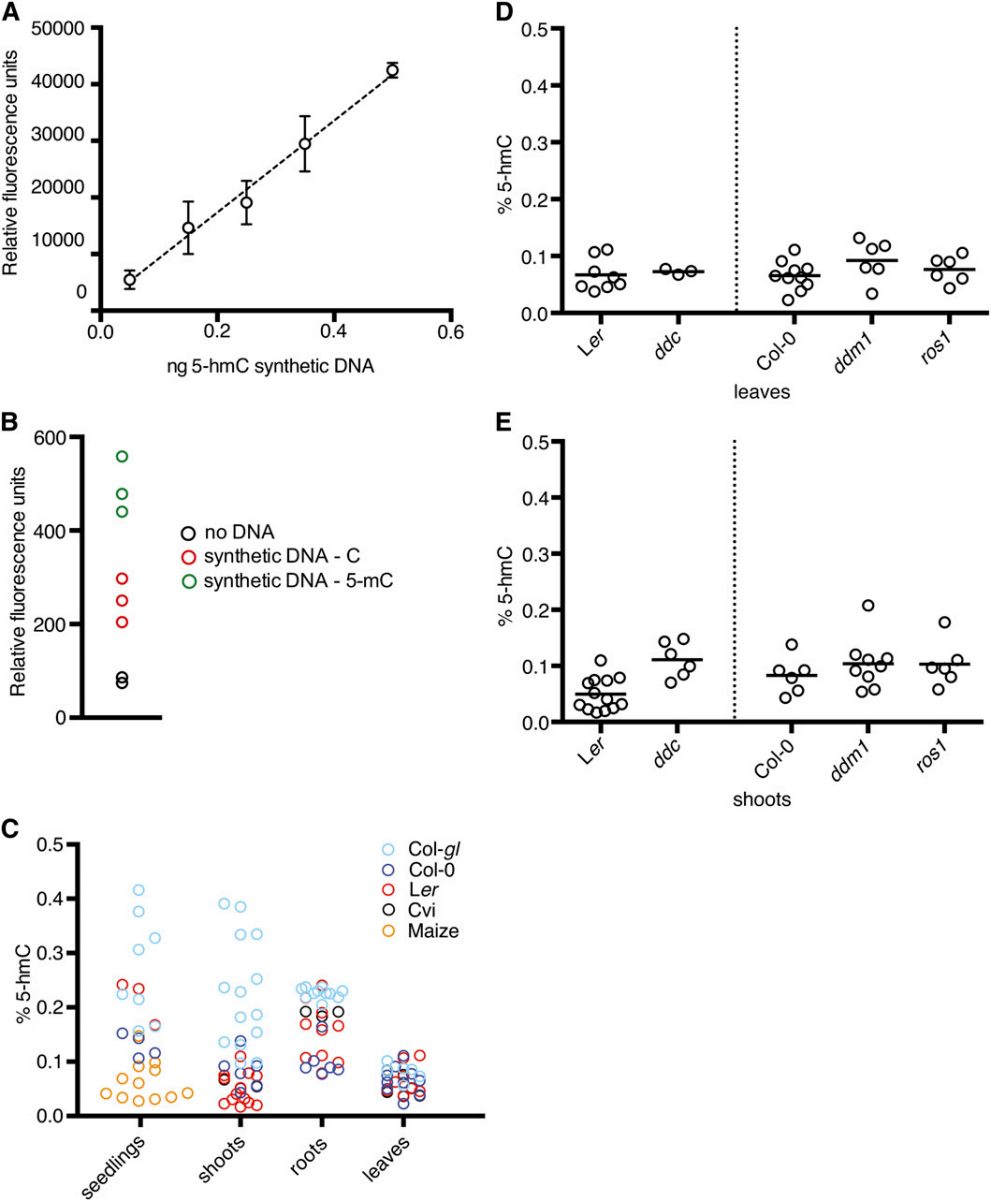
5-hmC is reproducibly measured at low concentrations in *Arabidopsis* by ELISA. (A) Example standard curve constructed using various amounts of 5-hmC–containing synthetic DNA. Error bars indicate SD of four technical replicates. (B) Negative control samples drawn from the same experiment as depicted in (A); 2 ng of synthetic DNA were used in each instance. (C) Quantity of 5-hmC calculated for *Arabidopsis* and maize tissue samples as percentage of total cytosines. (D, E) Percentage of 5-hmC calculated for leaves and shoots of various *Arabidopsis* methylation or demethylation mutants, compared with the wild-type strain of origin. Each circle represents an independent measurement. Horizontal bars indicate mean values.

To confirm these results and more precisely determine the level of 5-hmC in *Arabidopsis* DNA, we performed ELISA experiments against 5-hmC on a wide range of control and *Arabidopsis* DNA samples using a commercially available kit. Figure 3A illustrates the sensitivity of the method with a representative standard curve. Signal from 0.05 ng of 5-hmC–containing synthetic DNA, which corresponds to less than 0.1% 5-hmC in this experiment, was easily detected. Negative control samples had much lower signal when compared with 5-hmC–containing samples, with somewhat more signal from 5-mC than from C negative controls (Figure 3B). When applied to several different tissues from a variety of wild-type strains and *Zea mays*, the average tissue 5-hmC levels ranged from 0.07% to 0.17% of total cytosines (Figure 3C).

We also assayed the concentration of 5-hmC in a set of *Arabidopsis* DNA methylation and demethylation mutants, including *drm1 drm2 cmt3* (*ddc*), which is deficient in the *de novo* DNA methyltransferases, *ddm1*, deficient in a chromatin remodeling enzyme that is required to maintain DNA methylation in heterochromatin, and *ros1*, which is deficient in one of the 5-mC DNA glycosylases. Because 5-hmC is an oxidation product of 5-mC, it might be expected that mutants with reduced 5-mC (*ddc* and *ddm1*) would also be deficient in 5-hmC compared with wild-type, or that hypermethylation mutants (*ros1*) might have increased 5-hmC levels. These mutants had similar levels of 5-hmC signal when compared with wild-type strains and did not show significant variation when compared with each other (Figure 3D). Altogether, these results suggested the presence of a low but appreciable level of 5-hmC in plant DNA. However, because the 5-hmC antibodies might not be entirely specific to 5-hmC, as seen in Figure 2A, we sought additional confirmation of these results.

### Sensitive chemical assays do not directly detect 5-hmC in *Arabidopsis* DNA

**Figure 4:**
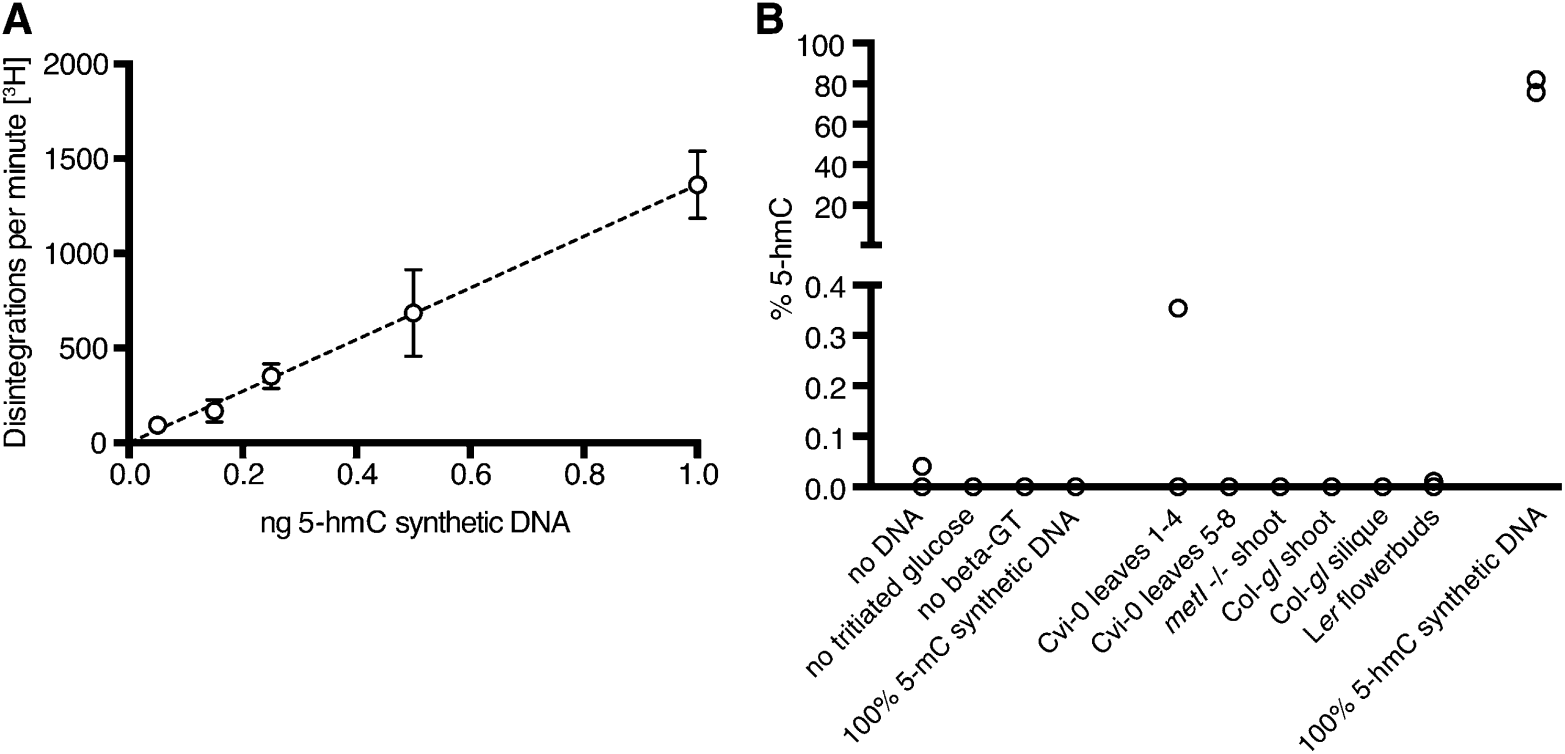
Radiolabeling assay does not detect 5-hmC in *Arabidopsis* genomic DNA. (A) Example standard curve constructed using various amounts of 5-hmC–containing synthetic DNA. (B) Percentage of 5-hmC calculated for negative controls (left), *Arabidopsis* genomic DNA samples (center), and positive controls (right). Each negative control sample was measured in triplicate, whereas each *Arabidopsis* sample was measured in duplicate. For both negative control samples and experimental samples, 92% of samples gave a reading of less than 0.01% 5-hmC.

We utilized a sensitive enzymatic labeling system to specifically radiolabel 5-hmC (Szwagierczak *et al.* 2010). The enzyme β-glucosyltransferase adds a glucose moiety to 5-hmC at the hydroxyl position but cannot modify 5-mC. Thus, the assay cleanly discriminates 5-mC and 5-hmC. Use of tritium-labeled glucose in the enzymatic reaction allows detection of 5-hmC. Figure 4A shows a representative standard curve from one of the experiments. The relationship between the disintegrations per minute and the amount of 5-hmC input was linear even at extremely low concentrations of 5-hmC—control samples with 0.01% 5-hmC were easily detected. When applying this method to genomic DNA from several *Arabidopsis* tissues and strains, including some genotypes that were assayed by ELISA (Figure 3), only one sample (a replicate of Cvi-0 leaves 1–4) out of 12 measured higher than 0.01% 5-hmC, with 10 of the samples yielding no detectable signal (Figure 4B). This is comparable with the results from the negative controls, where only 1 out of 12 samples (a no input DNA control) provided a reading more than 0.01% 5-hmC. Of note, controls with large quantities of 5-mC synthetic DNA never yielded a detectable signal, attesting to the lack of cross-reactivity with 5-mC.

Finally, we performed tandem mass spectrometry with the intention of unambiguously showing the presence or absence of 5-hmdC down to trace levels within *Arabidopsis* genomic DNA. Tandem mass spectrometry differentiates between closely related molecular species through changes in chromatography retention time, molecular weight measurements, and secondary fragment weight measurements, giving us the ability to simultaneously assay the relative quantities of dC, 5-mdC, and 5-hmdC for each sample. Table 1 illustrates the output of one set of mass spectrometry runs. Detection of 5-hmdC from positive controls, including 5-hmdC synthetic DNA and mouse cortex and ES cell DNA, was robust. Mouse cortex samples had greater 5-hmdC content than ES cell samples, as expected (Szwagierczak *et al.* 2010). Negative controls, both biological (*S. cerevisiae* genomic DNA) (Capuano *et al.* 2014) and synthetic (dC and 5-mdC–containing synthetic DNA), showed no evidence of 5-hmdC. Interestingly, the commercial 5-mdC and 5-hmdC nucleotides preparations were shown by mass spectrometry to not be completely pure for the expected nucleotide species. This is a good illustration of the power of mass spectrometry to detect sample impurities and distinguish between closely related species. The impurities do not impact the conclusions from any of the other assays. The *Arabidopsis* samples tested included seedling, flower bud, whole seed, and pollen DNA. The latter two sample types are known to lose DNA methylation during development (Gehring *et al.* 2009a; Hsieh *et al.* 2009; Calarco *et al.* 2012; Ibarra *et al.* 2012), and thus might be expected to harbor higher quantities of 5-hmdC if it was present and part of an alternative DNA demethylation pathway in the plant. None of the *Arabidopsis* tissue samples tested showed any trace of 5-hmdC, whereas the relative levels of dC and 5-mdC in the *Arabidopsis* samples agreed with expectations. The same results were obtained from additional mass spectrometry experiments (Figure S3).

**Table 1:** Relative abundance of deoxycytidines in *Arabidopsis* L*er* tissues and controls measured using LC-MS

Sample ID	dC (228.1 → 112.1)	5-mdC (242.2 → 126.1)	5-hmdC (258.2 → 142.1)
Yeast genomic DNA	6.04 × 10^4^	Not detected	Not detected
Mouse cortex DNA	7.61 × 10^5^	2.43 × 10^4^	2.57 × 10^3^
Mouse ES cell DNA	6.75 × 10^5^	1.90 × 10^4^	6.31 × 10^2^
*Arabidopsis* pollen DNA	5.34 × 10^5^	2.25 × 10^4^	Not detected
*Arabidopsis* whole seed DNA	1.02 × 10^6^	4.86 × 10^4^	Not detected
*Arabidopsis* seedling DNA	9.90 × 10^5^	5.91 × 10^4^	Not detected
*Arabidopsis* flower bud DNA	1.44 × 10^6^	9.14 × 10^4^	Not detected
dC PCR product	1.29 × 10^6^	4.93 × 10^2^	Not detected
5-mdC PCR product	1.54 × 10^5^	1.04 × 10^6^	Not detected
5-hmdC PCR product	2.82 × 10^5^	1.11 × 10^3^	5.41 × 10^5^
5-mdC nucleotides	4.42 × 10^2^	2.40 × 10^7^	8.63 × 10^2^
5-hmdC nucleotides	3.96 × 10^6^	6.82 × 10^4^	2.40 × 10^7^

## Discussion

We used assays with different sensitivities and specificities, including TLC, enzymatic labeling, immunoprecipitation, and mass spectrometry, to determine whether 5-hmC is present in plant DNA. Overall, the results indicate that 5-hmC is not present in significant quantities in *Arabidopsis*.

TLC is an excellent method for resolving closely related chemical species and was utilized in one of the first observations of 5-hmC within mammalian genomic DNA (Kriaucionis and Heintz 2009). Although our protocol had sufficient resolution to separate 5-hmdC from the more abundant dC and 5-mdC nucleotides, it had a lower limit of sensitivity on the order of tenths of a percent. Our TLC experiments on their own could not exclude the presence of 5-hmC in *Arabidopsis* genomic DNA, but instead set an upper value of less than 0.5% 5-hmC. The β-glucosyltransferase radiolabeling and mass spectrometry experiments had the greatest sensitivity of the assays we used. With changes to the original radiolabeling protocol (Szwagierczak *et al.* 2010), we were able to assay levels of 5-hmC less than 0.01% of cytosines, or less than 10 pg of 5-hmC, and we were unable to detect the base in *Arabidopsis*. Similarly, when using mass spectrometry, a gold standard method to identify rare molecular species within a complex sample, we were unable to detect 5-hmdC within a range of *Arabidopsis* samples, including seeds and pollen, which are hypomethylated and thus might be enriched for 5-hmdC if present (Gehring *et al.* 2009a, Hsieh *et al.* 2009, Ibarra *et al.* 2012, Calarco *et al.* 2012). The combination of these two results led us to conclude that 5-hmC is not present in biologically relevant quantities in *Arabidopsis* DNA.

In contrast, antibody-based methods yielded positive results for 5-hmC. However, these data are likely confounded by cross-reactivity. For the slot blot experiments, there was detectable cross-reactivity with the 5-mC control DNA under our conditions (Figure 2). 5-mC represents a sizeable fraction of cytosines within the *Arabidopsis* genome (Table 1) and it is possible that a portion of the signal from the plant tissue blots was actually 5-mC and not 5-hmC. The same issue of cross-reactivity could have affected the IP-chip experiment and the ELISA. As might be expected if the immunoprecipitated regions did not represent true signal, the 5-hmC profiling experiment had a somewhat lower dynamic range than the 5-mC profiling experiment. The detection of regions with high putative 5-hmC/low 5-mC and low 5-hmC/high 5-mC is puzzling when taken in the context of other experiments that fail to show even low levels of 5-hmC. These might represent nonspecific cross-reactivity of the different 5-hmC antibodies due to the lack of an appropriate antigen. The ELISA negative controls showed that 5-mC yields a signal approximately twice as strong as C (Figure 3B), meaning that 5-mC could have played a nontrivial role in the ELISA signal yielded by plant DNA.

One study reporting the presence of 5-hmC in *Arabidopsis* used only the antibody-based dot blot method (Yao *et al.* 2012). The percentage of 5-hmC reported in that study (∼0.07%) agrees well with some of the percentages we obtained through our ELISA experiments (Figure 3C). We believe that this concurrence emphasizes the need for caution in applying antibody-based methods for 5-hmC detection without the application of a secondary detection method, especially when 5-hmC is expected to be a rare species. Other studies that have reported on 5-hmC using nonantibody methods have concluded that 5-hmC is not present in biologically relevant amounts (Liu *et al.* 2013; Jang *et al.* 2014). Our study, which used a wide range of methods, strongly supports that conclusion.

## Supplementary Material

Supporting Information
