# Supplementary material for: 5-Hydroxymethylcytosine Is Not Present in Appreciable Quantities in Arabidopsis DNA
Source: G3 (Bethesda). 2014 Nov 6;5(1):1–8. doi: 10.1534/g3.114.014670 (PMC4291460; doi:10.1534/g3.114.014670)
Supplement: Supporting Information [file supp_5_1_1__index.html]

5-Hydroxymethylcytosine Is Not Present in Appreciable Quantities in Arabidopsis DNA — Supporting Information 

# 5-Hydroxymethylcytosine Is Not Present in Appreciable Quantities in *Arabidopsis* DNA

## Supporting Information for Erdmann *et al.*, 2015

**Files in this Data Supplement:**

- Supporting Information - Figures S1-S3 (PDF, 624 KB)
- Figure S1 - Control TLC plates illustrating threshold of 5-hmdC detection. (PDF, 712 KB)
- Figure S2 - IP-chip analysis and validation. (PDF, 452 KB)
- Figure S3 - Tandem LC-MS fails to detect 5-hmdC in *Arabidopsis* genomic DNA. (PDF, 643 KB)
