# Supplementary material for: 5-Hydroxymethylcytosine Is Not Present in Appreciable Quantities in Arabidopsis DNA
Source: G3 (Bethesda). 2014 Nov 6;5(1):1–8. doi: 10.1534/g3.114.014670 (PMC4291460; doi:10.1534/g3.114.014670)
Supplement: Supporting Information [file supp_g3.114.014670_014670SI.pdf]

G3: Genes | Genomes | Genetics

5-hydroxymethylcytosine is not present in appreciable quantities in *Arabidopsis* DNA

Robert M. Erdmann,* Amanda L. Souza,[§,1] Clary B. Clish,[§] Mary Gehring*[,2]

*Whitehead Institute, Cambridge, MA 02142 and Department of Biology, Massachusetts Institute of Technology, Cambridge, MA 02139, §Broad Institute, Cambridge, MA 02142

[1] Current address: Thermo Fisher Scientific, West Palm Beach, FL 33407

[2] Corresponding author: Whitehead Institute, 9 Cambridge Center, Cambridge, MA 02142; (617) 324-0343; mgehring@wi.mit.edu

DOI: 10.1534/g3.114.014670

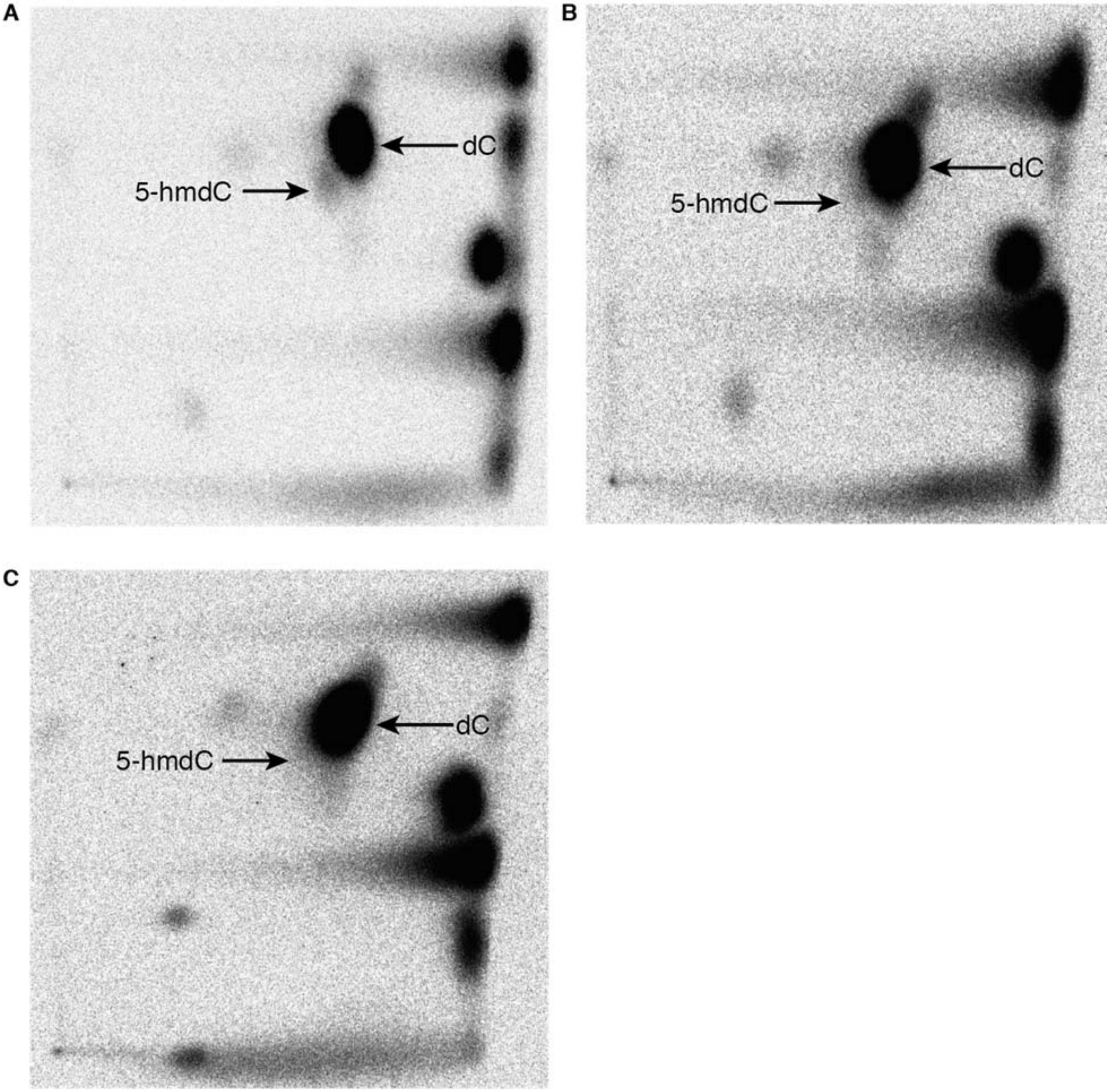

Figure S1 Control TLC plates illustrating threshold of 5-hmdC detection. (A) Plate spotted with 100 ng mixture of 95% dC and 5% 5-hmdC synthetic DNA. (B) Plate spotted with 100 ng mixture of 99.5% dC and 0.5% 5-hmdC synthetic DNA. (C) Plate spotted with 100 ng mixture of 99.9% dC and 0.1% 5-hmdC synthetic DNA. The expected location of 5-hmdC is labeled in (C), but at this concentration a distinct spot cannot be seen.

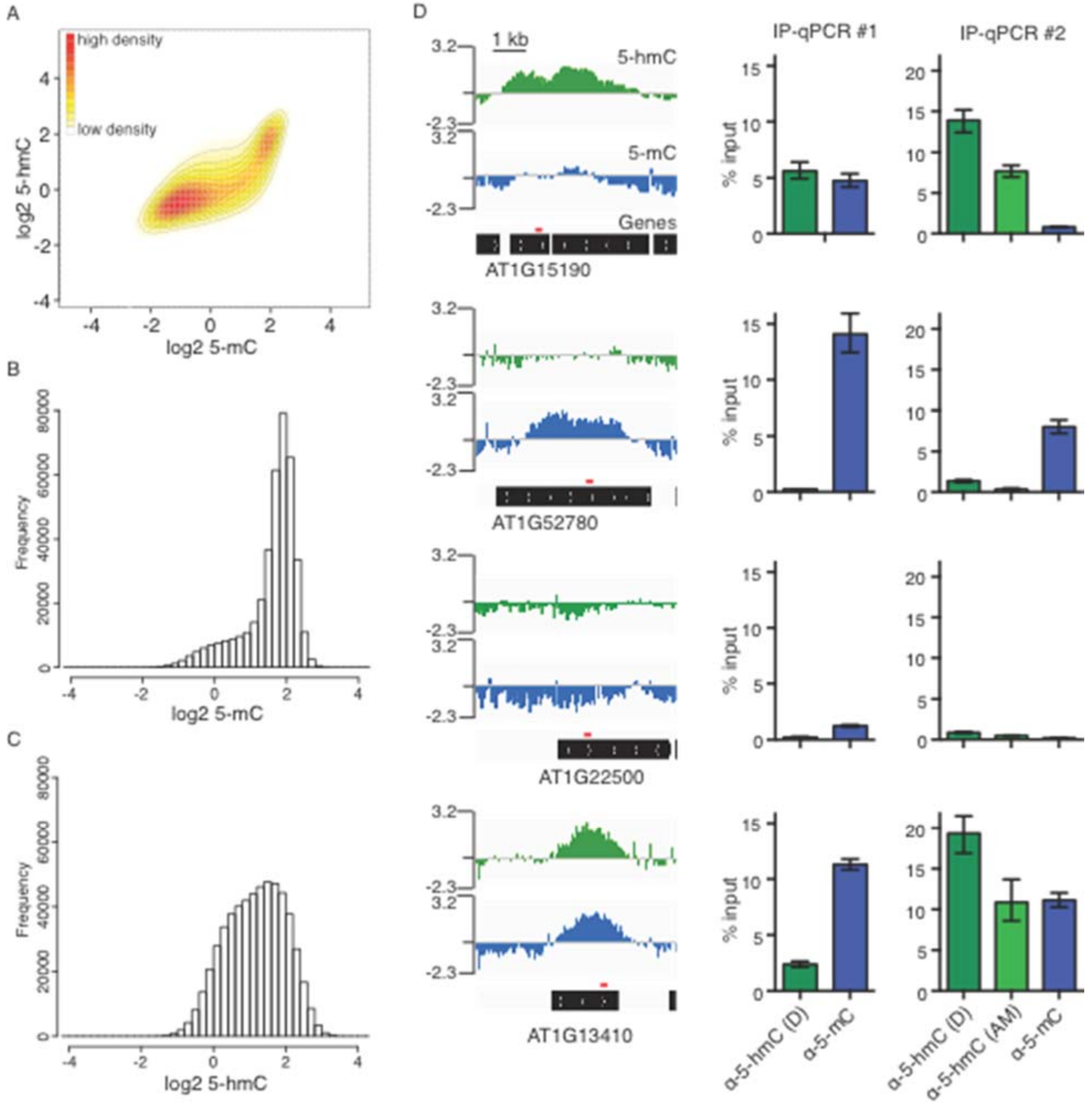

Figure S2 IP-chip analysis and validation. A) Contour plot illustrating the relative density of log2 5-mC vs. log2 5-hmC signal points. B) Histogram showing the distribution of log2 5-mC values for probes with a log2 5-hmC value greater than or equal to 1. C) Histogram showing the distribution of log2 5-hmC values for probes with a log2 5-mC value greater than or equal to 1. D) Validation of IP-chip by IP-qPCR. Four regions were chosen for validation (green tracks, 5-hmC IP-chip; blue tracks, 5-mC IP-chip; red bars, location of qPCR amplicons). In IP #1, 1.6 µg of sheared DNA from Col-0 flower buds was immunoprecipitated with 4.5 µg of either an α-5-hmC rat monoclonal antibody (Diagenode) or an α-5mC mouse monoclonal antibody (Diagenode). In IP #2, 4 µg of sheared DNA from Col-0 flower buds was immunoprecipitated with 10 µg of either the α-5-hmC rat monoclonal antibody (Diagenode), an α-5-hmC rabbit polyclonal antibody (Active Motif), or the α-5mC mouse monoclonal antibody (Diagenode). IP-qPCR results are expressed as the percent of input DNA. Error bars represent standard deviation of three technical replicates. IP-qPCR #2 used the same input and antibody amounts as the IP-chip experiment, except that the commercial source of the α-5mC mouse monoclonal antibody was different.

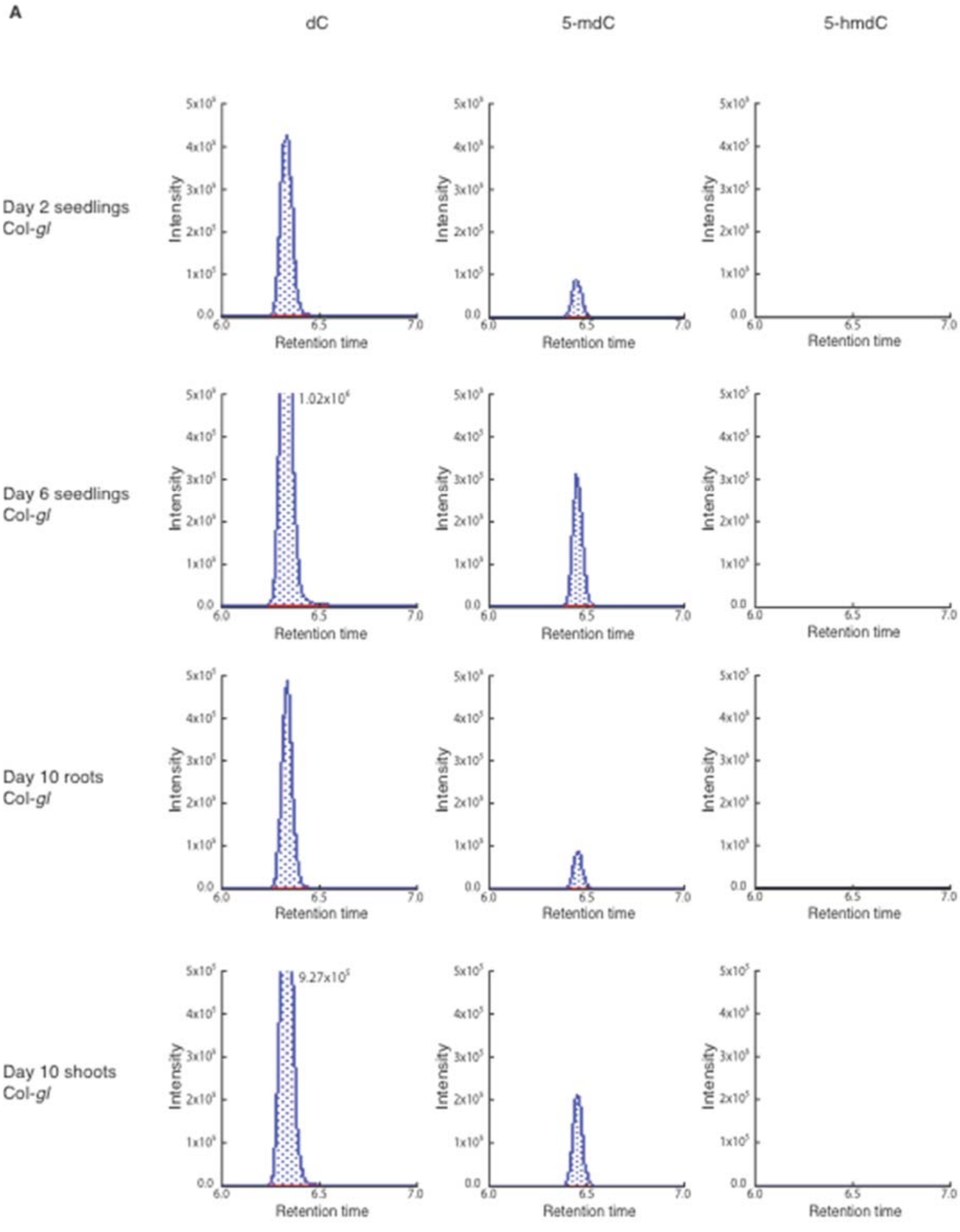
A
dC
5-mdC
5-hmdC
Day 2 seedlings
Col-gl
Day 6 seedlings
Col-gl
Day 10 roots
Col-gl
Day 10 shoots
Col-gl
Intensity
Retention time
1.02x10⁶
9.27x10⁵

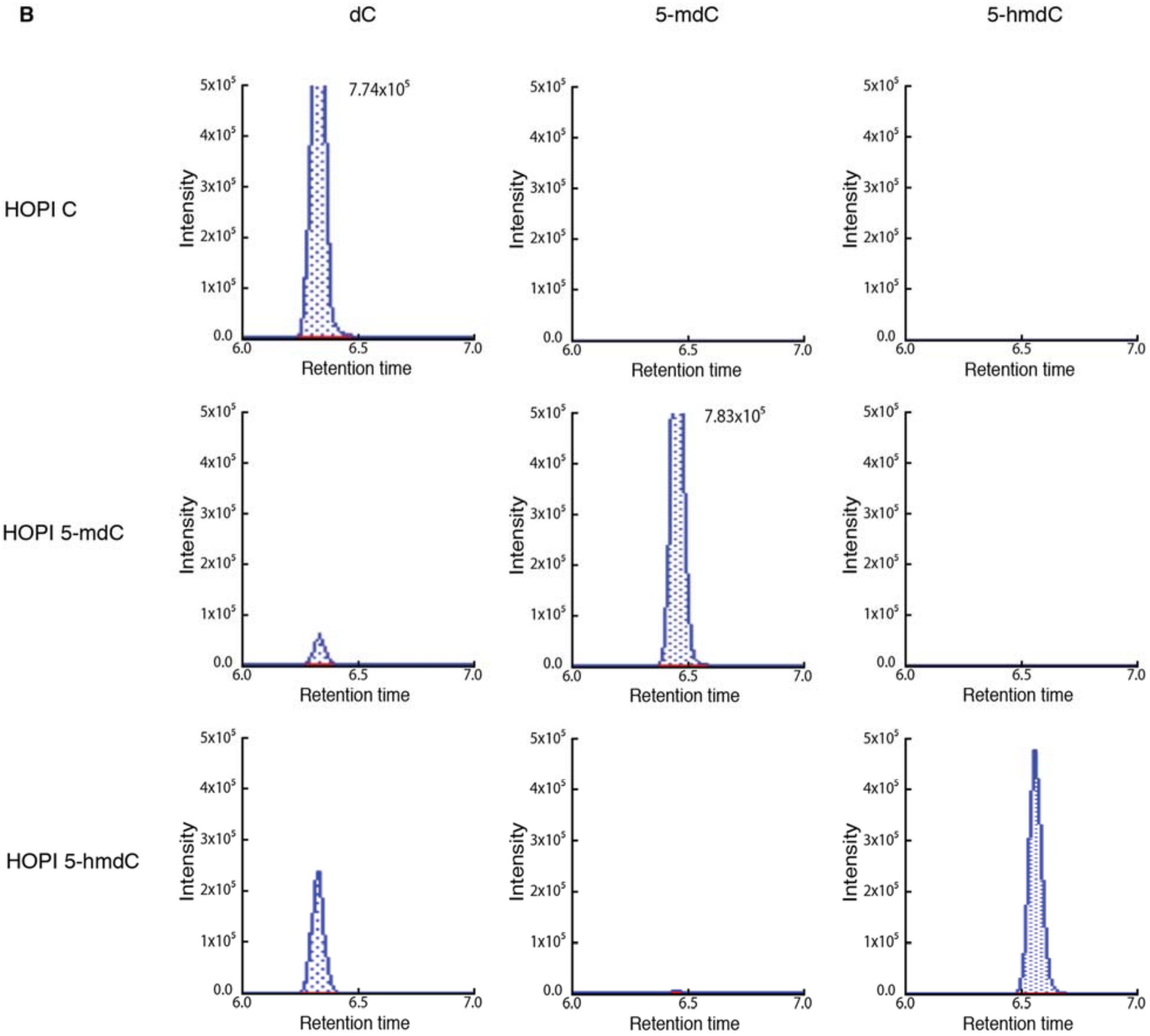
B
dC
5-mdC
5-hmdC
HOPI C
HOPI 5-mdC
HOPI 5-hmdC
7.74x10⁵
7.83x10⁵
Intensity
Retention time

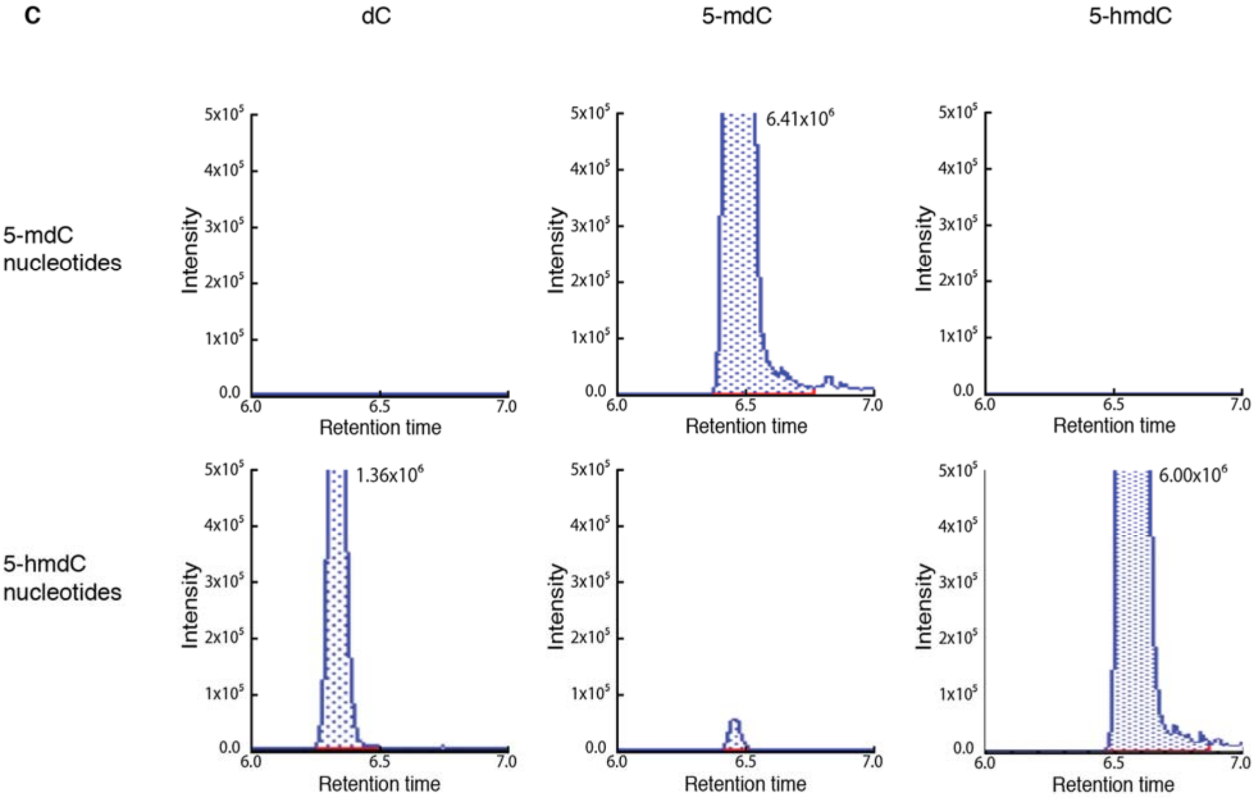

Figure S3 Tandem LC-MS fails to detect 5-hmdC in *Arabidopsis* genomic DNA. (A) dC, 5-mdC, and 5-hmdC levels in genomic DNA from *Arabidopsis* seedling tissues. dC and 5-mdC levels hold similar ratios to each other in all four tissues assayed, but none of the tissues show a 5-hmdC peak. (B) dC, 5-mdC, and 5-hmdC levels in synthetic DNA controls. (C) dC, 5-mdC, and 5-hmdC levels in source nucleotide samples. 5-hmdC nucleotides show a sizeable amount of dC signal. For peaks that go above the top of the intensity scale, the number label indicates the actual height of the peak. Retention time unit is minutes.
