# Supplementary material for: 5-Hydroxymethylcytosine Is Not Present in Appreciable Quantities in Arabidopsis DNA
Source: G3 (Bethesda). 2014 Nov 6;5(1):1–8. doi: 10.1534/g3.114.014670 (PMC4291460; doi:10.1534/g3.114.014670)
Supplement: Supporting Information [file supp_g3.114.014670_FigureS1.pdf]

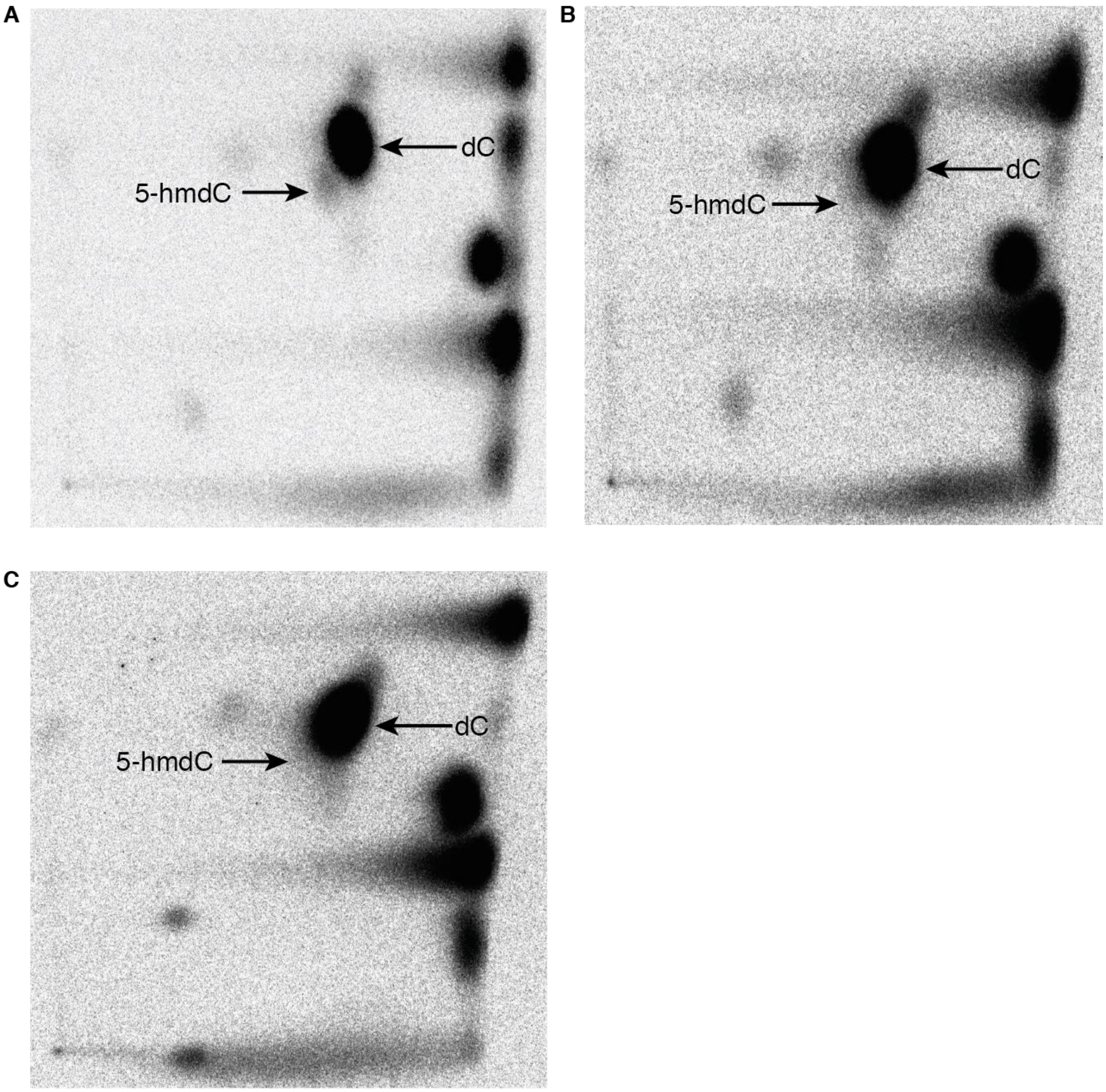

Figure S1 Control TLC plates illustrating threshold of 5-hmdC detection. (A) Plate spotted with 100 ng mixture of 95% dC and 5% 5-hmdC synthetic DNA. (B) Plate spotted with 100 ng mixture of 99.5% dC and 0.5% 5-hmdC synthetic DNA. (C) Plate spotted with 100 ng mixture of 99.9% dC and 0.1% 5-hmdC synthetic DNA. The expected location of 5-hmdC is labeled in (C), but at this concentration a distinct spot cannot be seen.
