# Supplementary material for: 5-Hydroxymethylcytosine Is Not Present in Appreciable Quantities in Arabidopsis DNA
Source: G3 (Bethesda). 2014 Nov 6;5(1):1–8. doi: 10.1534/g3.114.014670 (PMC4291460; doi:10.1534/g3.114.014670)
Supplement: Supporting Information [file supp_g3.114.014670_FigureS2.pdf]

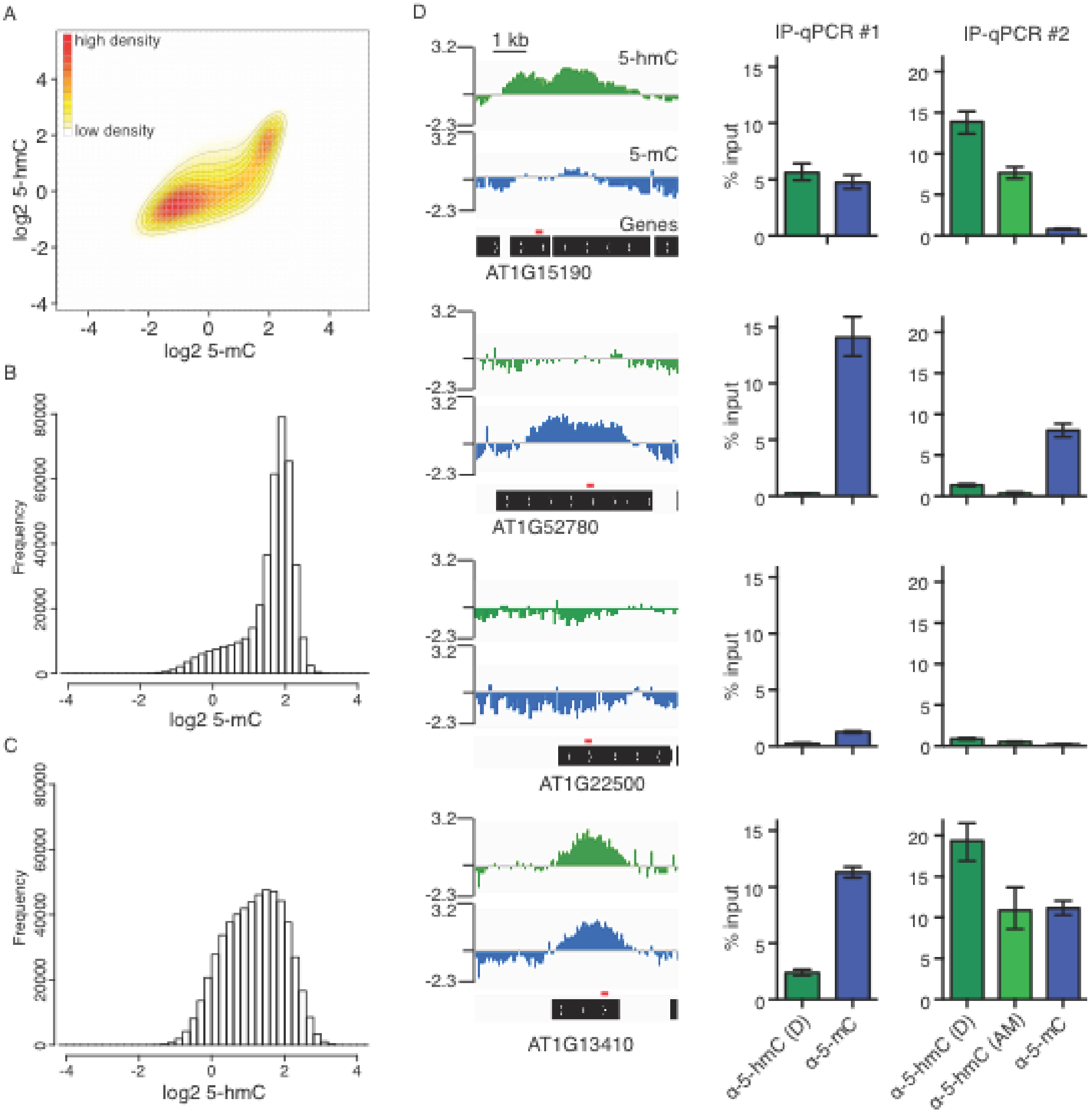

Figure S2 IP-chip analysis and validation. A) Contour plot illustrating the relative density of log2 5-mC vs. log2 5-hmC signal points. B) Histogram showing the distribution of log2 5-mC values for probes with a log2 5-hmC value greater than or equal to 1. C) Histogram showing the distribution of log2 5-hmC values for probes with a log2 5-mC value greater than or equal to 1. D) Validation of IP-chip by IP-qPCR. Four regions were chosen for validation (green tracks, 5-hmC IP-chip; blue tracks, 5-mC IP-chip; red bars, location of qPCR amplicons). In IP #1, 1.6 µg of sheared DNA from Col-0 flower buds was immunoprecipitated with 4.5 µg of either an α-5-hmC rat monoclonal antibody (Diagenode) or an α-5mC mouse monoclonal antibody (Diagenode). In IP #2, 4 µg of sheared DNA from Col-0 flower buds was immunoprecipitated with 10 µg of either the α-5-hmC rat monoclonal antibody (Diagenode), an α-5-hmC rabbit polyclonal antibody (Active Motif), or the α-5mC mouse monoclonal antibody (Diagenode). IP-qPCR results are expressed as the percent of input DNA. Error bars represent standard deviation of three technical replicates. IP-qPCR #2 used the same input and antibody amounts as the IP-chip experiment, except that the commercial source of the α-5mC mouse monoclonal antibody was different.
