# Supplementary material for: 5-Hydroxymethylcytosine Is Not Present in Appreciable Quantities in Arabidopsis DNA
Source: G3 (Bethesda). 2014 Nov 6;5(1):1–8. doi: 10.1534/g3.114.014670 (PMC4291460; doi:10.1534/g3.114.014670)
Supplement: Supporting Information [file supp_g3.114.014670_FigureS3.pdf]

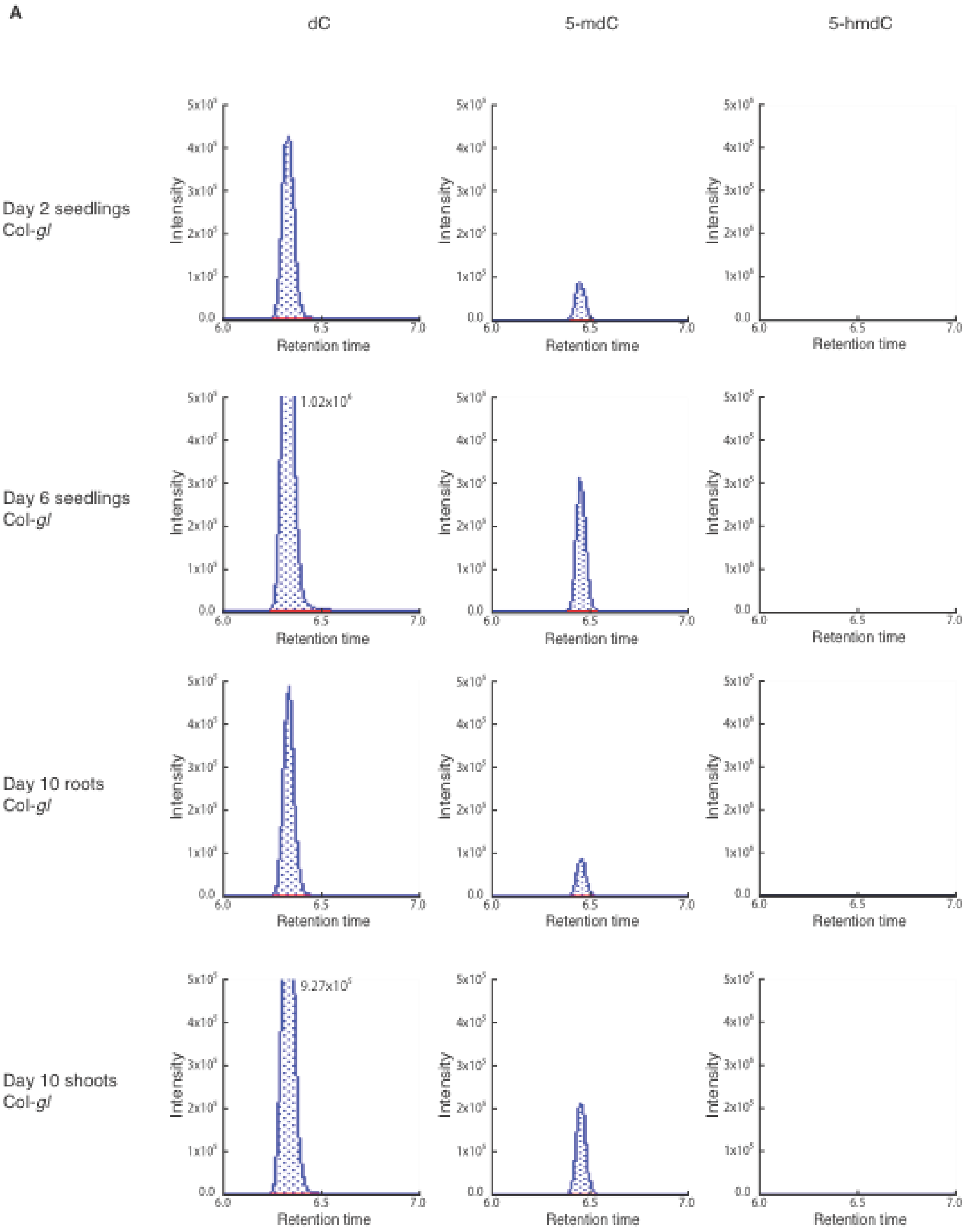

A
dC
5-mdC
5-hmdC
Day 2 seedlings
Col-gl
Day 6 seedlings
Col-gl
Day 10 roots
Col-gl
Day 10 shoots
Col-gl
Intensity
Retention time
1.02x10^6
9.27x10^5

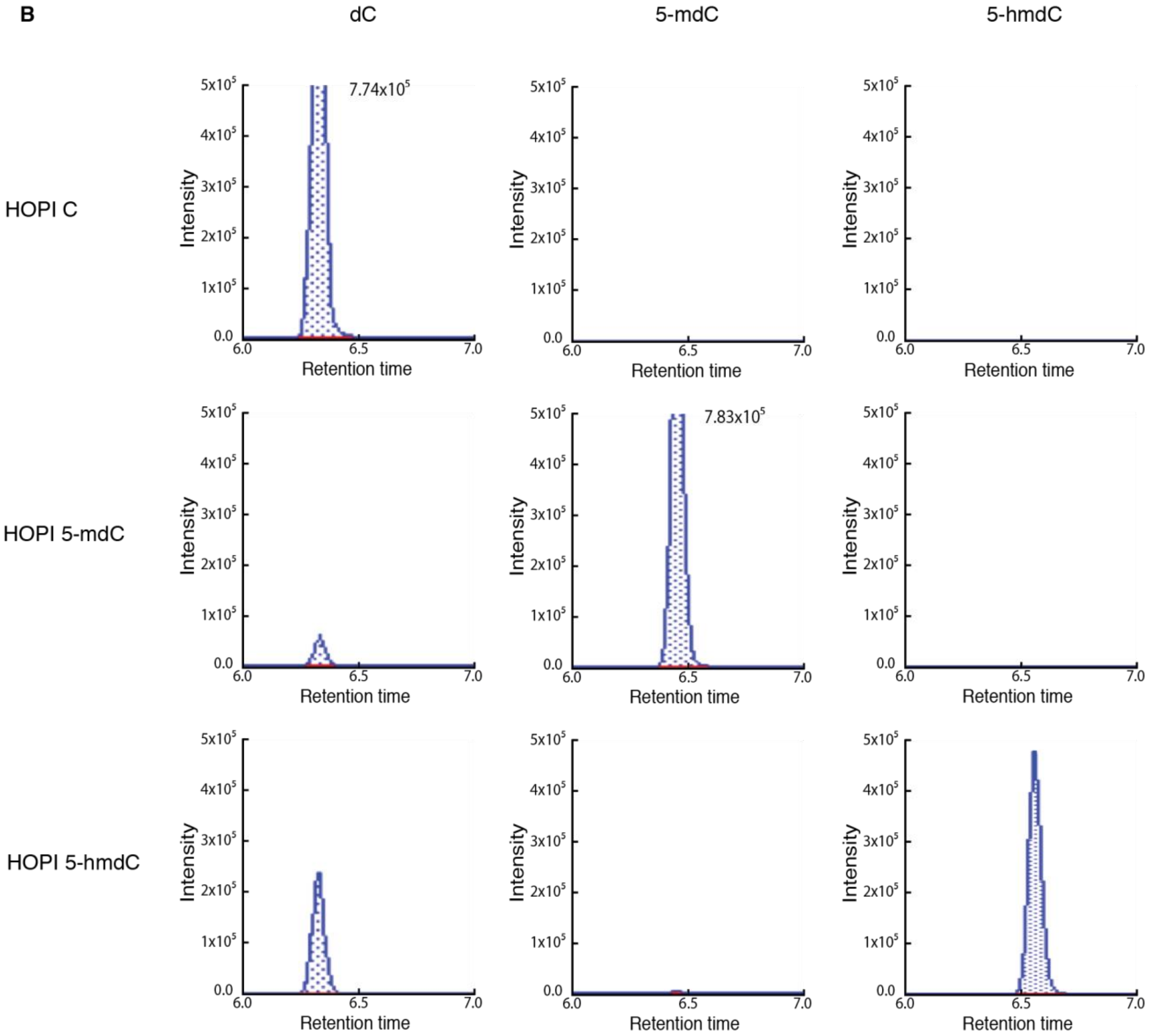
B
dC
5-mdC
5-hmdC
HOPI C
HOPI 5-mdC
HOPI 5-hmdC
7.74x10^5
7.83x10^5
Intensity
Retention time

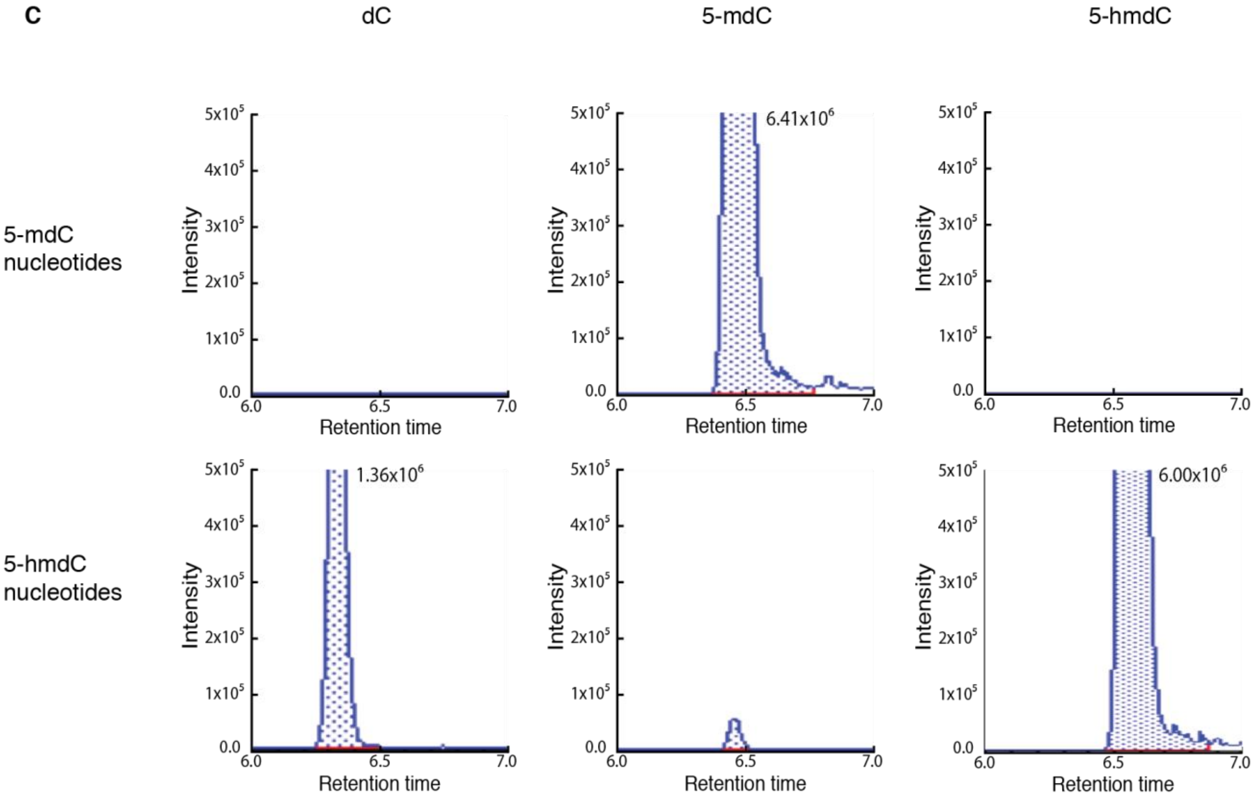

Figure S3 Tandem LC-MS fails to detect 5-hmdC in *Arabidopsis* genomic DNA. (A) dC, 5-mdC, and 5-hmdC levels in genomic DNA from *Arabidopsis* seedling tissues. dC and 5-mdC levels hold similar ratios to each other in all four tissues assayed, but none of the tissues show a 5-hmdC peak. (B) dC, 5-mdC, and 5-hmdC levels in synthetic DNA controls. (C) dC, 5-mdC, and 5-hmdC levels in source nucleotide samples. 5-hmdC nucleotides show a sizeable amount of dC signal. For peaks that go above the top of the intensity scale, the number label indicates the actual height of the peak. Retention time unit is minutes.
